# Implementation of a Model of Bodily Fluids Regulation

**DOI:** 10.1007/s10441-015-9250-3

**Published:** 2015-05-03

**Authors:** Julie Fontecave-Jallon, S. Randall Thomas

**Affiliations:** CNRS, TIMC-IMAG Laboratory CNRS UMR 5525, PRETA Team, University Joseph Fourier-Grenoble 1, 38041 Grenoble, France; IR4M UMR8081 CNRS, University Paris-Sud, Orsay, France

**Keywords:** Computational physiology, Acid–base balance, Mathematical modelling, Virtual physiological human (VPH)

## Abstract

The classic model of blood pressure regulation by Guyton et al. (Annu Rev Physiol 34:13–46, 1972a; Ann Biomed Eng 1:254–281, 1972b) set a new standard for quantitative exploration of physiological function and led to important new insights, some of which still remain the focus of debate, such as whether the kidney plays the primary role in the genesis of hypertension (Montani et al. in Exp Physiol 24:41–54, 2009a; Exp Physiol 94:382–388, 2009b; Osborn et al. in Exp Physiol 94:389–396, 2009a; Exp Physiol 94:388–389, 2009b). Key to the success of this model was the fact that the authors made the computer code (in FORTRAN) freely available and eventually provided a convivial user interface for exploration of model behavior on early microcomputers (Montani et al. in Int J Bio-med Comput 24:41–54, 1989). Ikeda et al. (Ann Biomed Eng 7:135–166, 1979) developed an offshoot of the Guyton model targeting especially the regulation of body fluids and acid–base balance; their model provides extended renal and respiratory functions and would be a good basis for further extensions. In the interest of providing a simple, useable version of Ikeda et al.’s model and to facilitate further such extensions, we present a practical implementation of the model of Ikeda et al. (Ann Biomed Eng 7:135–166, 1979), using the ODE solver Berkeley Madonna.

**Electronic supplementary material:**

The online version of this article (doi:10.1007/s10441-015-9250-3) contains supplementary material, which is available to authorized users.

## Introduction

Computational modelling in physiology has contributed to many significant breakthroughs, but the models themselves have usually not become working tools for experimentalists nor even for other modellers outside the developer’s own group. We provide here a practical implementation of one of the classic and most complete models of body fluid and acid–base regulation, and we give several examples of the use of the model. We give the complete model description in the language of Berkeley Madonna, which is very easy to read and can readily be converted for other numerical solvers. Physiologists and clinicians will find this model easy to use, and this complete example will facilitate extensions in order to simulate related clinical situations or new experimental findings.

Inspired by the classic model of blood pressure regulation by Guyton et al. (1972a), Ikeda et al. (1979) adopted the same symbolic representation to illustrate model structure, but since their focus was on body fluids and acid–base balance, which have a slower time course than, say, autonomic regulation of cardiovascular variables, they simplified the representation of the cardiovascular system but greatly extended the renal and respiratory systems. Their model consists of a set of nonlinear differential and algebraic equations with more than 200 variables and has subsystems for circulation, respiration, renal function, and intra- and extra-cellular fluid spaces.

## Materials and Methods

### Model Description

The original article of Ikeda et al. (1979) describes the details of the model, so we will not give a complete description here (the program code, Online Resource 01, given in the Electronic Supplementary Material and described in the Appendix, has all the explicit equations); our implementation closely follows the description in their article, especially in their diagrams of the seven blocks that constitute the model, namely, the circulation and body fluids (blocks 1, 3, and 4), respiration (block 2), and renal function (blocks 5, 6, and 7). Initial values and many other details are given not only in the text but also on the diagrams and in the tables of the original article. Here, we give just a brief explanation of the basic content of the model and Ikeda et al.’s general strategy.

 As in Ikeda et al. (1979), the model assumes a healthy male of approximately 55 kg body weight, and parameter values used here are those given in the original article. Calibration of the model for other body weights or for females would be a valuable extension of the model but is beyond the goals of the present work. Such extension would involve re-calibration not only of extracellular and intracellular fluid volumes (and thus with impact on solute contents of those compartments), but also of less straightforward parameters such as metabolic rate, respiratory volume, cardiac output, and the like.

The cardiovascular/circulatory (CV) system, quite complex in Guyton’s model, was considerably simplified by Ikeda et al. (1979) to a simple steady state that represents the system’s state after settling from transient local autoregulation or stress relaxation.

By contrast with the simplified CV system, and in keeping with their focus on acid–base and fluid physiology, Ikeda et al. (1979) included much more elaborate representations of the respiratory system, intracellular and extracellular electrolytes and solutes, and of course the kidney. For example:

- Alveolar ventilation (VI) is calculated as a function of blood pH, $\hbox {P}_{\hbox{CO}_{2}}$, and $\hbox {P}_{\hbox{O}_{2}}$;
- The blood buffer system is treated using the Henderson–Hasselbalch equation, an equation for the oxygen saturation curve, and an equation for the in vivo $\hbox{CO}_{2}$ dissociation curve, thus the model takes account of the haemoglobin buffer system, the Bohr effect, and the Haldane effect;
- The model treats intra- and extra-cellular electrolytes and acid–base balance and also glucose metabolism and insulin secretion—disorders of glucose metabolism can be modelled by varying the parameters CGL1, CGL2 and CGL3;
- Plasma osmolality in the model depends on the concentrations not only of sodium, potassium, glucose, and urea, but also of mannitol, included in the model because of its frequent therapeutic use;
- The renal blocks treat reabsorption and excretion not only of water, sodium, and potassium, but also of bicarbonate, calcium, magnesium, phosphate, and organic acids; proximal tubule reabsorption depends on volume expansion or pressure diuresis (THDF); aldosterone is assumed to act on the distal tubule to increase sodium reabsorption, decrease potassium secretion, and increase excretion of titratable acid; urine pH and excretion of ammonia, titratable acid, phosphate, and organic acids are included in the model; glomerular filtration rate (GFR), represented as a sigmoid function of arterial pressure, is controlled by extracellular volume (VEC) and depends on antidiuretic hormone (ADH) and aldosterone (ALD) and on THDF;
- The renin–angiotensin–aldosterone system (RAAS) is represented here simply as a transfer function by which ALD secretion depends on extracellular fluid (ECF) potassium concentration, tubular sodium concentration, arterial pressure, and volume receptor signals.

In addition to this incomplete list, the model contains many other interesting features that the reader should glean from the original Ikeda et al. (1979) article.

#### Berkeley Madonna Description

 Berkeley Madonna is a fast, robust, multi-platform solver of systems of ordinary differential-algebraic equations. Compared with other such solvers, it is extremely easy to program (a simple list of the equations in any order), has a very effective user interface for plotting or tabulating the results and varying the parameters (simple “sliders” can be easily defined to vary individual model variables or parameters, with instant re-run of the model), and it has proven to be very fast compared to other solvers we have used.

## Results

To demonstrate several interesting features of the model and also to show that the Berkeley Madonna implementation presented here is an accurate representation of the Ikeda et al. model, we show that it faithfully reproduces the results of four simulations whose results are shown in the figures of their article. The BM codes used to generate the results of the following simulations are all provided as Electronic Supplementary Material (see Appendix).

**Fig. 1:**
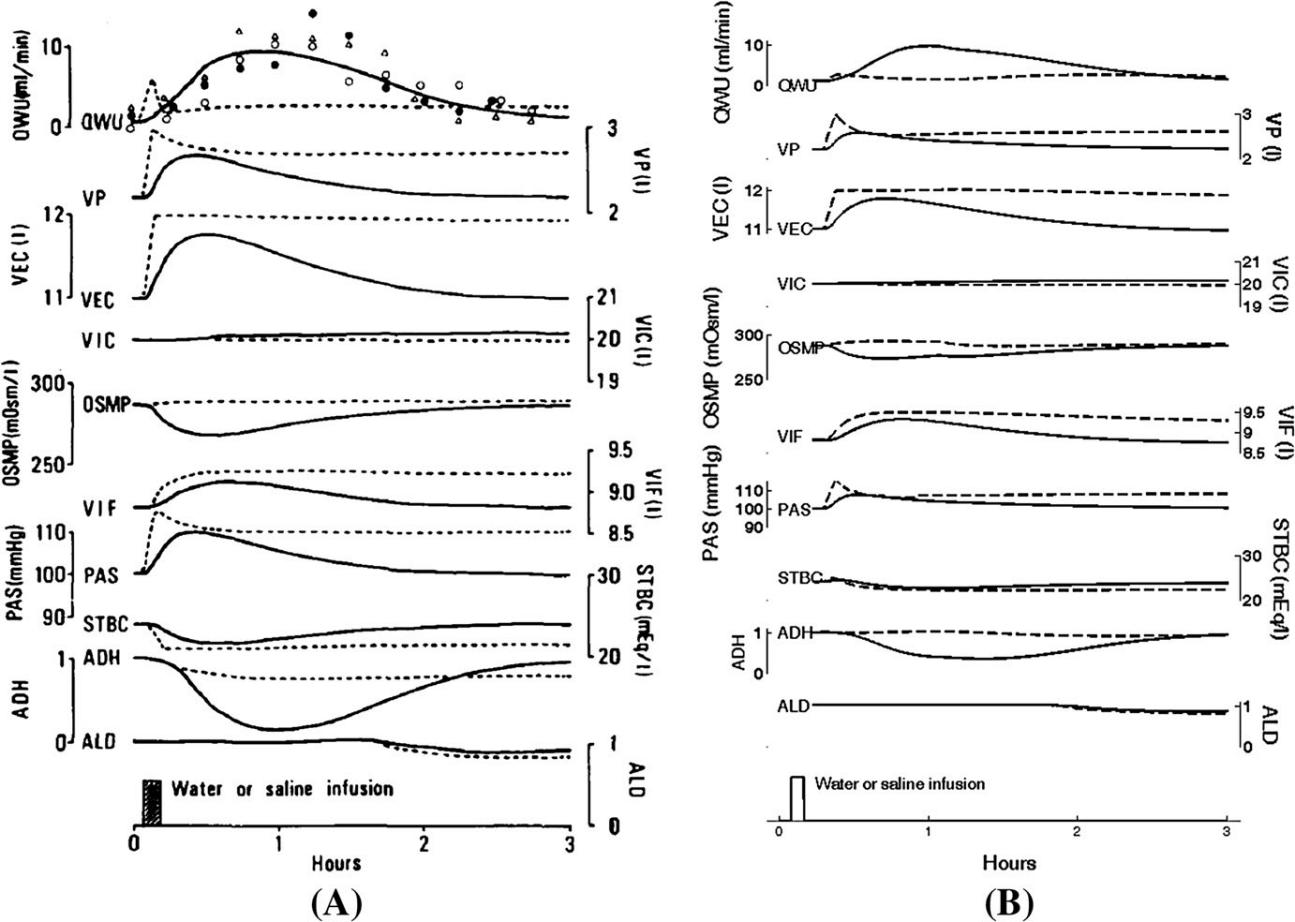
**a** Simulation of oral water intake (*solid lines*) and intravenous infusion of physiological saline (*dashed lines*), both at a rate of 1000 ml per 5 min (see Fig. 10 in Ikeda et al. (1979)). **b** The same simulations were carried out in Berkeley-Madonna. We simulate, during 3 h, the responses of body fluid and kidney parameters to acute water loading (*solid lines*) at a rate of 200 ml/min during 5 min (rate of drinking, QIN=0.2 l/min from t = 5 to 10 min) and to intravenous normal saline infusion (*dashed lines*), solution of 0.9 % w/v of NaCl, containing 154 mEq/l of $\hbox {Na}^+$ and $\hbox {Cl}^-$, at the same rate during 5 min (from t = 5 to 10 min, the rate of intravenous water input was QVIN = 0.2 l/min , and intake rate of sodium and chloride was YNIN = YCLI = 30.8 mEq/min). For the simulation of oral water intake (Online Resource 02), the user must replace the following line of BM code: QIN = 0.001 with: QIN = IF (TIME $\ge$ 5 AND TIME $\le$ 10) THEN 0.2 ELSE 0.001. For the simulation of intravenous infusion of physiological saline (Online Resource 03), the user must replace the following lines of BM code: QVIN = 0, YCLI = 0.1328 and YNIN = 0.12 with: QVIN = IF (TIME $\ge$ 5 AND TIME $\le$ 10) THEN 0.2 ELSE 0, YCLI = IF (TIME $\ge$ 5 AND TIME $\le$ 10) THEN 154*0.2 ELSE 0.1328, YNIN = IF (TIME $\ge$ 5 AND TIME $\le$ 10) THEN 154*0.2 ELSE 0.12. We observe the rate of urinary output (QWU), the plasma volume (VP), the volume of extracellular fluid (VEC), the intracellular fluid volume (VIC), the plasma osmolality (OSMP), the interstitial fluid volume (VIF), the systemic arterial pressure (PAS), the standard bicarbonate at pH = 7.4 (STBC), the effect of antidiuretic hormone (ADH), and the effect of aldosterone (ALD)

Figure 1 shows the results of a simulation of oral water intake (1 l over 5 min) and intravenous infusion of physiological saline; the left panel shows Fig. 10 from the Ikeda article, and the right panel shows results from our BM model, which are clearly a good match to those in their article.

**Fig. 2:**
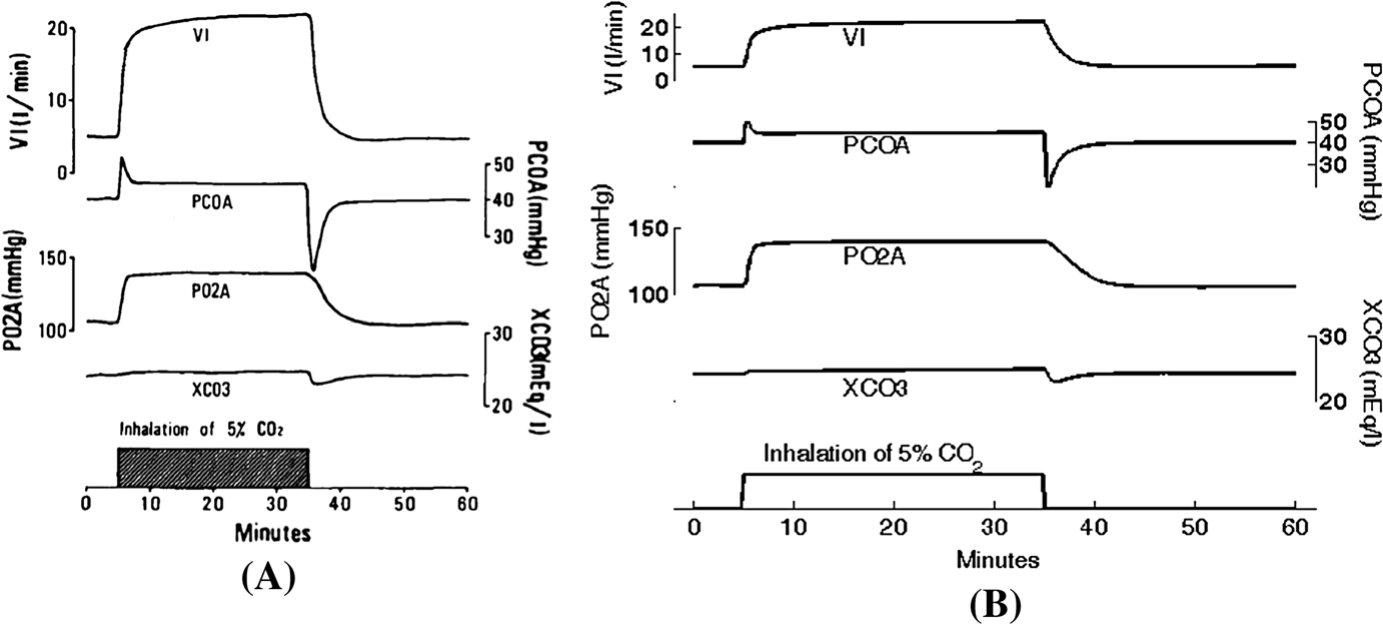
**a** Simulation of the transient response of the respiratory system to 5 % $\hbox {CO}_2$ inhalation (see Fig. 11 in Ikeda et al. Ikeda et al. (1979)). **b** The same simulation was carried out in Berkeley-Madonna (Online Resource 04). We simulate, during 1 h, the transient response of the respiratory parameters to the inhalation of 5 % $\hbox {CO}_2$ in air over 30 min (volume fraction of $\hbox {CO}_2$ in dry inspired gas FCOI = 0.05 from t = 5 to 35 min). The user must replace the following line of BM code: FCOI = 0 with: FCOI = IF (TIME $\ge$ 5 AND TIME $\le$ 35) THEN 0.05 ELSE 0. We observe the alveolar ventilation (VI), the pressure of $\hbox {CO}_2$ and $\hbox {O}_2$ in the alveoli (PCOA and PO2A), and the concentration of bicarbonate of the extracellular fluid (XCO3)

Figure 2 shows the transient response of respiratory parameters to inhalation of 5 % $\hbox {CO}_2$ over 30 minutes; the left panel shows Fig. 11 from the Ikeda article, and the right panel shows results from our BM model.

**Fig. 3:**
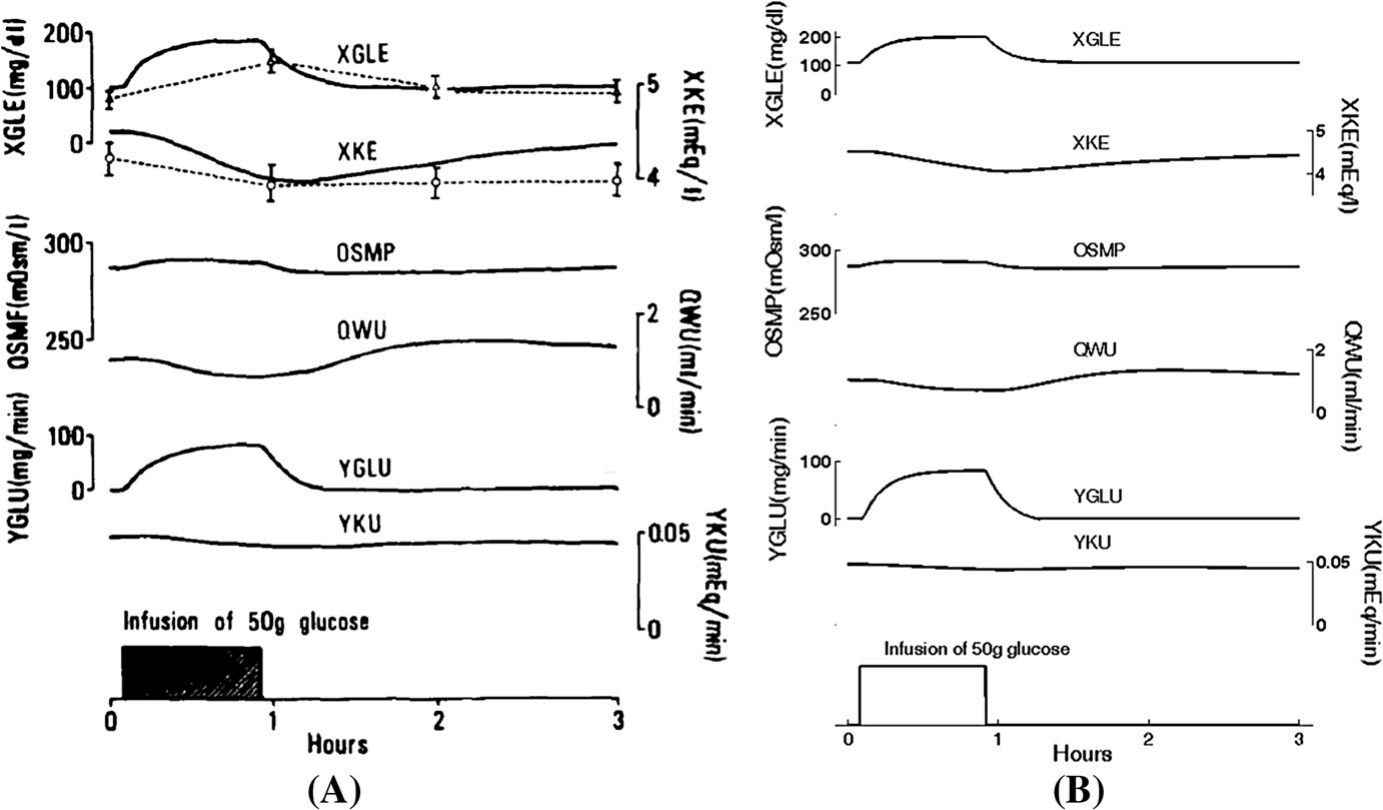
**a** Simulation (Fig. 12 in Ikeda et al. Ikeda et al. (1979)) of the glucose tolerance curve with the extracellular fluid potassium concentration. **b** The same simulation was carried out in Berkeley-Madonna (Online Resource 05). We simulate, during 3 h, a test of glucose metabolism, corresponding to the infusion of glucose at a rate of 1 g/min during 50 min (intake rate of glucose YGLI = 1000 from t = 5 to t = 55 min). The user must replace the following line of the BM code: YGLI = 0 with: YGLI = IF (TIME $\ge$ 5 AND TIME $\le$ 55) THEN 1000 ELSE 0. We observe the ECF glucose concentration (XGLE), the ECF potassium concentration (XKE), the plasma osmolality (OSMP), the rate of urinary output (QWU), the renal excretion of glucose (YGLU), and the rate of renal loss of potassium (YKU)

Figure 3 shows results from a simulation of glucose tolerance test (infusion of 50 g of glucose over 1 h), including insulin secretion due to a concomitant decrease of extracellular fluid potassium concentration; as above, the left panel shows Fig. 12 from the Ikeda article, and the right panel shows the corresponding results from our BM model.

**Fig. 4:**
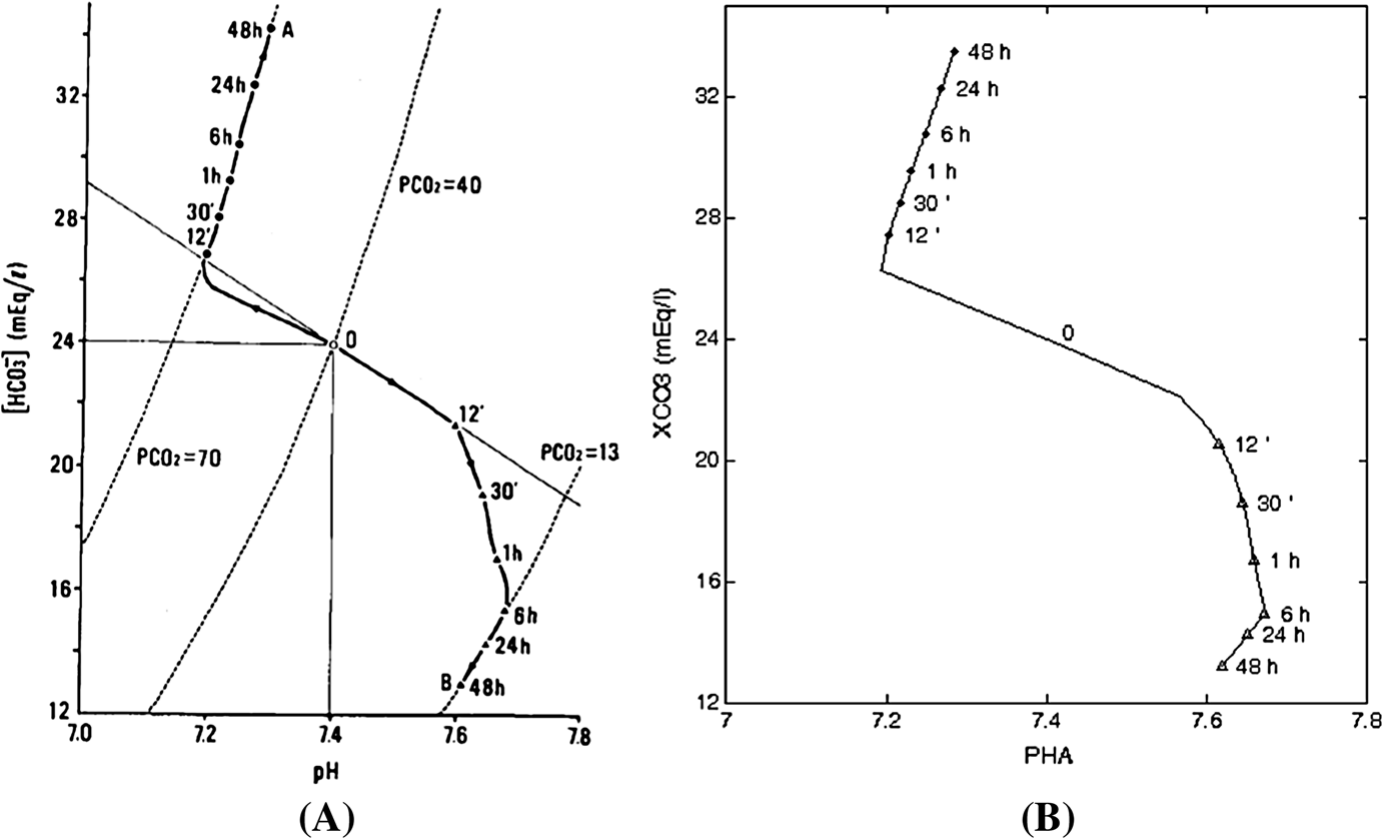
**a** Simulation (Fig. 13 in Ikeda et al. Ikeda et al. (1979)) of respiratory acidosis and alkalosis with renal compensation. Point *O* shows the normal value of the model of the pH-[$\hbox {HCO}_3$] plane. *Triangle* indicates the plotting of simulated response to 10 % $\hbox {CO}_2$ inhalation for 48 h, and *Filled circle* indicates that of hyperventilation, in which VI was fixed at 15 1/min. Equi-pressure lines of $\hbox {PCO}_2$ are shown with *dotted lines* for the $\hbox {PCO}_2$ values of 13.3, 40.0, and 73.0  mmHg. **b** The same simulations were carried out in Berkeley-Madonna. We first simulate (Online Resource 06), during 48  h, the response to 10 % $\hbox {CO}_2$ inhalation (volume fraction of $\hbox {CO}_2$ in dry inspired gas FCOI at the value of 0.1, rather than 0, during the whole simulation and equation (1) unmodified). The bicarbonate concentration of the extracellular fluid (XCO3) and the pH of arterial blood (PHA) are measured at various times from 12 min to 48 h and plotted with *Triangle* line. We then simulate (Online Resource 07) during 48 h the response to hyperventilation, in which VI was raised to three times normal (alveolar ventilation VI is kept constant to 15  l/min, VI=15, replacing equation (1) of the BM code during the whole simulation). The volume fraction of $\hbox {CO}_2$ in dry inspired gas FCOI is set at its normal value 0. XCO3 and PHA are measured at various times from 12 min to 48 h and plotted with *Filled circle* line

Figure 4 shows, in acid–base disturbances, the central role of the kidney in the compensatory reactions of the body when the normal response of respiration does not occur. The long-term time course of the model behavior in respiratory acidosis or alkalosis is depicted on the pH-[$\hbox {HCO}_{3}$] diagram. The response to 10 % $\hbox {CO}_2$ inhalation and the response to hyperventilation are observed. The right panel shows the results from our BM model, which are in good agreement with the results of Ikeda article, shown on the left panel. The sequence of steps necessary to reproduce this figure with BM implementation is detailed in the specific BM code listing (Online Resources 06 & 07).

## Discussion

Efforts towards reusability and interoperability have made progress in recent years, not only in the modeling of kidney physiology (Thomas 2009) but also in the wider context of physiology and systems biology (Hunter et al. 2013). For instance, SBML (the Systems Biology Markup language)^1^ (Hucka et al. 2003) is widely used for metabolic networks and models of cell signal transduction, the CellML repository^2^ contains several hundred marked-up legacy models (mostly at the level of membrane transport or signal transduction), the JSim Consolidated Model Database^3^ indexes 73390 models across five archives, and annotation tools such as the RICORDO^4^ resource (de Bono et al. 2011) and the ApiNATOMY^5^ (de Bono et al. 2012) project now facilitate the sharing (and even the merging) of physiology and systems biology models.

The present work complements previous re-implementations of the Ikeda model; e.g., a Pascal version was used in teaching at the University of Limburg, Maastricht (Min (1982); Pascal source code in Min (1993)), and extensions of parts of the Ikeda model were used in the Golem simulator (Kofranek et al. 2001). The present Berkeley Madonna version also complements our re-implementations of the early Guyton models (Hernandez et al. 2011; Moss et al. 2012; Thomas et al. 2008) and recent models focused on the kidney itself (Karaaslan et al. 2005, 2014; Moss et al. 2009; Moss and Thomas 2014) or on the role of the kidney in blood pressure regulation (Averina et al. 2012; Beard and Mescam 2012). We provide here a convenient implementation of the Ikeda et al. (1979) model in order to facilitate not only its use in its original form but also to enable its extension. One such improvement would be the incorporation of a more complete model of the RAAS system, which is now much better understood and for which a detailed model has recently been published (Guillaud and Hannaert 2010).

### Electronic supplementary material

Supplementary material 1 (pdf 89 KB)

Supplementary material 2 (pdf 36 KB)

Supplementary material 3 (pdf 36 KB)

Supplementary material 4 (pdf 37 KB)

Supplementary material 5 (pdf 37 KB)

Supplementary material 6 (pdf 36 KB)

Supplementary material 7 (pdf 37 KB)

Supplementary material 8 (pdf 37 KB)

## Appendix

### Program Code

The source code for our implementation of the model of Ikeda et al. (1979), using the ODE solver Berkeley Madonna, is available as Supplementary Material on the website of Acta Biotheoretica. In addition to the basic version that corresponds strictly to the description in the original article, we also provide variants used to produce the figures of the present article.

We release the model codes under the CeCill free software license agreement (a copy of the CeCill free software license agreement is included as Online Resource 00, file: ESM_00).

We provide the following Berkeley Madonna source code files:

1. Online Resource 01 (file: “ESM_01”): Basic code for simulation of the model in steady-state (file: “ESM_01”)
2. Oral water intake and intravenous infusion of physiological saline (Fig. 10 of Ikeda et al. (1979))
  - Online Resource 02, file: “ESM_02”—Simulation of water intake at a rate of 1000 ml per 5 min.
  - Online Resource 03, file: “ESM_03”—Simulation of intravenous infusion of physiological saline at a rate of 1000 ml per 5 min.
3. Transient response of the respiratory system to 5 % $\hbox {CO}_2$ inhalation (Fig. 11 of Ikeda et al. (1979))
  - Online Resource 04, file: “ESM_04”—Simulation of the inhalation of 5 % $\hbox {CO}_2$ in air over 30 min.
4. Glucose tolerance curve with the extracellular potassium concentration (Fig. 12 of Ikeda et al. (1979))
  - Online Resource 05, file: “ESM_05”—Simulation during 3 h of a test of glucose metabolism, corresponding to the infusion of glucose at a rate of 1 g/min during 50 min.
5. Respiratory acidosis and alkalosis with renal compensation (Fig. 13 of Ikeda et al. (1979)).
  - Online Resource 06, file: “ESM_06”—Simulation of 10 % $\hbox {CO}_2$ inhalation during 48 h.
  - Online Resource 07, file: “ESM_07”—Simulation of ventilation at 15 l/min during 48 h.

### List of Variables

Here we give the table of variables, with units and normal or initial values.

STPD refers to “standard temperature and pressure, dry”, denoting a volume of dry gas at 0 $^{\circ }$C and a pressure of 760 mmHg.

**Table:**

Symbol	Definition	Normal value
ADH	Effect of antidiuretic hormone (ratio to normal)	1
ALD	Effect of aldosterone (ratio to normal)	1
CFC	Capillary filtration coefficient	0.007 l/min/mmHg
CGL1	Parameter of glucose metabolism	1
CGL2	Parameter of glucose metabolism	1
CGL3	Parameter of glucose metabolism	0.03
CHEI	Transfer coefficient of hydrogen ion into ICF	5
CKAL	Weight of effect of XKE on aldosterone secretion	0.5
CNAL	Weight of effect on YNH on aldosterone secretion	0.1
CPAL	Weight of effect of PAS on aldosterone secretion	0.01
CPVL	Weight of effect of PVP on aldosterone secretion	0.1
COAD	Weight of effect of OSMP on ADH secretion	0.5
CPAD	Weight of effect of PVP on ADH secretion	1.0
CKEI	Potassium transfer coefficient from ECF to ICF	0.001
CPRX	Excretion ratio of filtered load after proximal tubule	0.2
CRAV	Arterial resistance/venous resistance	5.93
CSM	Transfer coefficient of water from ECF to ICF	0.0003 $\hbox {l}^2/\hbox {mEq}/\hbox {min}$
DCLA	Chloride shift	0 mEq/l
DEN	Proportional constant between QCO and VB	1
FCOA	Volume fraction of $\hbox {CO}_2$ in dry alveolar gas	0.0561
FCOI	Volume fraction of $\hbox {CO}_2$ in dry inspired gas	0
FO2A	Volume fraction of $\hbox {O}_2$ in dry alveolar gas	0.1473
FO2I	Volume fraction of $\hbox {O}_2$ in dry inspired gas	0.21
GFR	Glomerular filtration rate	0.1 l/min
GFR0	Normal value of GFR	0.1 l/min
HF0-HF4	Parameters related to the abnormal state of the heart	0
HT	Hematocrit	45 %
KL	Parameter of left heart performance	0.2
KR	Parameter of right heart performance	0.3
MRCO	Metabolic production rate of $\hbox {CO}_2$	0.2318 l(STPD)/min
MRO2	Metabolic production rate of $\hbox {O}_2$	0.2591 l(STPD)/min
OSMP	Plasma osmolality	287 mOsm/l
OSMU	Urine osmolality	461 mOsm/l
PAP	Pulmonary arterial pressure	20 mmHg
PAS	Systemic arterial pressure	100 mmHg
PBA	Barometric pressure	760 mmHg
PBL	PBA-Vapor pressure	713 mmHg
PC	Capillary pressure	17 mmHg
PCOA	$\hbox {CO}_2$ tension in alveoli	40 mmHg
PF	Filtration pressure	0.3 mmHg
PHA	pH of arterial blood	7.4
PHI	pH of intracellular fluid	7.0
PHU	pH of urine	6.0
PICO	Interstitial colloid osmotic pressure	5.0 mmHg
PIF	Interstitial fluid pressure	$-$6.3 mmHg
PO2A	$\hbox {O}_2$ tension in alveoli	105 mmHg
PPCO	Plasma colloid osmotic pressure	28 mmHg
PVP	Pulmonary venous pressure	4 mmHg
PVP0	Parameter of left heart performance	0 mmHg
PVS	Systemic venous pressure	3 mmHg
PVSO	Parameter of right heart performance	0 mmHg
QCFR	Capillary filtration rate	0.002 l/min
QCO	Cardiac output	5 l/min
QIC	Rate of water flow into intracellular space	0 l/min
QIN	Drinking rate	0.001 l/min
QIWL	Rate of insensible water loss	0.0005 l/min
QLF	Rate of lymph flow	0.02 l/min
QMWP	Rate of metabolic water production	0.0005 l/min
QPLC	rate of protein through capillary	0.000799 l/min
QVIN	Rate of intravenous water input	0 l/min
QWD	Rate of urinary excretion in distal tubule	0.01 l/min
QWU	Urine output	0.001 l/min
RTOP	Total resistance in pulmonary circulation	3 mmHg.min/l
RTOT	Total resistance in systemic circulation	20 mmHg.min/l
STBC	Standard bicarbonate at pH = 7.4	24 mEq/l
TADH	Time constant of ADH secretion	30 min
TALD	Time constant of aldosterone secretion	30 min
THDF	Effect of third factor (ratio to normal)	l
UCOA	Content of $\hbox {CO}_2$ in arterial blood	0.5612 l(STPD)/l.blood
UCOV	Content of $\hbox {CO}_2$ in venous blood	0.6075 l(STPD)/l.blood
UHB	Blood $\hbox {O}_2$ combining power	0.2 l.02 (STPD)/l.blood
UHBO	Blood oxyhemoglobin	0.2 l.02 (STPD)/l.blood
UO2A	Content of $\hbox {O}_2$ in arterial blood	0.2033 l(STPD)/l.blood
UO2V	Content of $\hbox {O}_2$ in venous blood	0.1515 l(STPD)/l.blood
VAL	Total alveolar volume	3 l
VB	Blood volume	4 l
VEC	Extracellular fluid volume	11 l
VI	Ventilation	5 l/min
VI0	Normal value of ventilation	5 l/min
VIC	Intracellular fluid volume	20 l
VIF	Interstitial fluid volume	8.8 l
VP	Plasma volume	2.2 l
VRBC	Volume of red blood cells	1.8 l/min
VTW	Total body fluid volume	31 l
XCAE	ECF calcium concentration	5 mEq/l
XCLA	Arterial chloride concentration	104 mEq/l
XCLE	ECF chloride concentration	104 mEq/l
XCO3	ECF bicarbonate concentration	24 mEq/l
XGL0	Reference value of ECF glucose concentration	108 mg/dl
XGLE	ECF glucose concentration	6 mg/l
XHB	Blood hemoglobin concentration	15 g/dl
XKE	ECF potassium concentration	4.5 mEq/l
XKI	ICF potassium concentration	140 mEq/l
XMGE	ECF magnesium concentration	3 mEq/l
XMNE	ECF mannitol concentration	0 mEq/l
XNE	ECF sodium concentration	140 mEq/l
XOGE	ECF organic acid concentration	6 mM/l
XPIF	Interstitial protein concentration	20 g/l
XPO4	ECF phosphate concentration	1.1 mM/l
XPP	Plasma protein concentration	70 g/l
XSO4	ECF sulphate concentration	1 mEq/l
XURE	ECF urea concentration	2.5 mM/l
YCA	Renal excretion rate of calcium	0.007 mEq/min
YCAI	Intake rate of calcium	0.007 mEq/min
YCLI	Intake rate of chloride	0.1328 mEq/min
YCLU	Renal excretion rate of chloride	0.1328 mEq/min
YCO3	Renal excretion rate of bicarbonate	0.015 mEq/min
YGLI	Intake rate of glucose	0 mg/min
YGLU	Renal excretion of glucose	0 mg/min
YINS	Intake rate of insulin	0 U/min
YKD	Rate of potassium excretion in distal tubule	0.1205 mEq/min
YKIN	Intake rate of potassium	0.047 mEq/min
YKU	Renal excretion rate of potassium	0.047 mEq/min
YMG	Renal excretion rate of magnesium	0.008 mEq/min
YMGI	Intake rate of magnesium	0.008 mEq/min
YMNI	Intake rate of mannitol	0 mM/min
YMNU	Renal excretion rate of mannitol	0 mM/min
YND	Rate of sodium excretion in distal tubule	1.17 mEq/min
YNH	Rate of sodium excretion in Henle loop	1.4 mEq/min
YNH0	Normal excretion rate of ammonium	0.024 mEq/min
YNH4	Renal excretion rate of ammonium	0.024 mEq/min
YNIN	Intake rate of sodium	0.12 mEq/min
YNU	Renal excretion rate of sodium	0.12 mEq/min
YOGI	Intake rate of organic acid	0.01 mM/min
YORG	Renal excretion rate of organic acid	0.01 mM/min
YPG	Flow of protein into interstitial gel	0 g/min
YPLC	Flow of protein through capillary	0.04 g/min
YPLF	Flow of protein in lymphatic vessel	0.04 g/min
YPLG	Flow of protein into pulmonary fluid	0 g/min
YPLV	Destruction rate of protein in liver	0 g/min
YPO4	Renal excretion rate of phosphate	0.025 mM/min
YPOI	Intake rate of phosphate	0.025 mM/min
YSO4	Renal excretion rate of sulphate	0.02 mEq/min
YSOI	Intake rate of sulphate	0.02 mEq/min
YTA	Renal excretion rate of titratable acid	0.0168 mEq/min
YTA0	Normal excretion rate of titratable acid	0.0068 mEq/min
YURI	Intake rate of urea	0.15 mM/min
YURU	Renal excretion rate of urea	0.15 mM/min
ZCAE	ECF calcium content	55 mEq
ZCLE	ECF chloride content	1144 mEq
ZGLE	ECF glucose content	66 mg
ZKE	ECF potassium content	49.5 mEq
ZKI	ICF potassium content	2800 mEq
ZMGE	ECF magnesium content	33 mEq
ZMNE	ECF mannitol content	0 mM
ZNE	ECF sodium content	1540 mEq
ZOGE	ECF organic acid content	66 mM
ZPG	Protein content in interstitial gel	20 g
ZPIF	ISF protein content	176 g
ZPLG	Protein content in pulmonary fluid	70 g
ZPO4	ECF phosphate content	12.1 mM
ZPP	Plasma protein content	154 g
ZSO4	ECF sulphate content	11 mEq
ZURE	ECF urea content	77.5 mM

## Footnotes

1 http://sbml.org.

2 https://models.cellml.org/cellml.

3 http://physiome.org/jsim/db/.

4 http://www.ricordo.eu.

5 http://apinatomy.org.
