# Supplementary material for: Implementation of a Model of Bodily Fluids Regulation
Source: Acta Biotheor. 2015 May 3;63(3):269–82. doi: 10.1007/s10441-015-9250-3 (PMC4531145; doi:10.1007/s10441-015-9250-3)
Supplement: Supplementary file 1 — Supplementary material 1 (pdf 89 KB) [file 10441_2015_9250_MOESM1_ESM.pdf]

CeCILL FREE SOFTWARE LICENSE AGREEMENT

Notice

This Agreement is a Free Software license agreement that is the result of discussions between its authors in order to ensure compliance with the two main principles guiding its drafting:

- firstly, compliance with the principles governing the distribution of Free Software: access to source code, broad rights granted to users,
- secondly, the election of a governing law, French law, with which it is conformant, both as regards the law of torts and intellectual property law, and the protection that it offers to both authors and holders of the economic rights over software.

The authors of the CeCILL[1] license are:

Commissariat à l'Energie Atomique - CEA, a public scientific, technical and industrial research establishment, having its principal place of business at 25 rue Leblanc, immeuble Le Ponant D, 75015 Paris, France.

Centre National de la Recherche Scientifique - CNRS, a public scientific and technological establishment, having its principal place of business at 3 rue Michel-Ange, 75794 Paris cedex 16, France.

Institut National de Recherche en Informatique et en Automatique - INRIA, a public scientific and technological establishment, having its principal place of business at Domaine de Voluceau, Rocquencourt, BP 105, 78153 Le Chesnay cedex, France.

Preamble

The purpose of this Free Software license agreement is to grant users the right to modify and redistribute the software governed by this license within the framework of an open source distribution model.

The exercising of these rights is conditional upon certain obligations for users so as to preserve this status for all subsequent redistributions.

In consideration of access to the source code and the rights to copy, modify and redistribute granted by the license, users are provided only with a limited warranty and the software's author, the holder of the economic rights, and the successive licensors only have limited liability.

In this respect, the risks associated with loading, using, modifying and/or developing or reproducing the software by the user are brought to the user's attention, given its Free Software status, which may make it complicated to use, with the result that its use is reserved for developers and experienced professionals having in-depth computer knowledge. Users are therefore encouraged to load and test the suitability of the software as regards their requirements in conditions enabling the security of their systems and/or data to be ensured and, more generally, to use and operate it in the same conditions of security. This Agreement may be freely reproduced and published, provided it is not altered, and that no provisions are either added or removed herefrom.

This Agreement may apply to any or all software for which the holder of the economic rights decides to submit the use thereof to its provisions.

Article 1 - DEFINITIONS

For the purpose of this Agreement, when the following expressions commence with a capital letter, they shall have the following meaning:

Agreement: means this license agreement, and its possible subsequent versions and annexes.

Software: means the software in its Object Code and/or Source Code form and, where applicable, its documentation, “as is” when the Licensee accepts the Agreement.

Initial Software: means the Software in its Source Code and possibly its Object Code form and, where applicable, its documentation, “as is” when it is first distributed under the terms and conditions of the Agreement.

Modified Software: means the Software modified by at least one Contribution.

Source Code: means all the Software’s instructions and program lines to which access is required so as to modify the Software.

Object Code: means the binary files originating from the compilation of the Source Code.

Holder: means the holder(s) of the economic rights over the Initial Software.

Licensee: means the Software user(s) having accepted the Agreement.

Contributor: means a Licensee having made at least one Contribution.

Licensor: means the Holder, or any other individual or legal entity, who distributes the Software under the Agreement.

Contribution: means any or all modifications, corrections, translations, adaptations and/or new functions integrated into the Software by any or all Contributors, as well as any or all Internal Modules.

Module: means a set of sources files including their documentation that enables supplementary functions or services in addition to those offered by the Software.

External Module: means any or all Modules, not derived from the Software, so that this Module and the Software run in separate address spaces, with one calling the other when they are run.

Internal Module: means any or all Module, connected to the Software so that they both execute in the same address space.

GNU GPL: means the GNU General Public License version 2 or any subsequent version, as published by the Free Software Foundation Inc.

Parties: mean both the Licensee and the Licensor.

These expressions may be used both in singular and plural form.

Article 2 - PURPOSE

The purpose of the Agreement is the grant by the Licensor to the Licensee of a non-exclusive, transferable and worldwide license for the Software as set forth in Article 5 hereinafter for the whole term of the protection granted by the rights over said Software.

Article 3 - ACCEPTANCE

3.1 The Licensee shall be deemed as having accepted the terms and conditions of this Agreement upon the occurrence of the first of the following events:

- (i) loading the Software by any or all means, notably, by downloading from a remote server, or by loading from a physical medium;
- (ii) the first time the Licensee exercises any of the rights granted hereunder.

3.2 One copy of the Agreement, containing a notice relating to the characteristics of the Software, to the limited warranty, and to the fact that its use is restricted to experienced users has been provided to the Licensee prior to its acceptance as set forth in Article 3.1 hereinabove, and the Licensee hereby acknowledges that it has read and understood it.

Article 4 - EFFECTIVE DATE AND TERM

4.1 EFFECTIVE DATE

The Agreement shall become effective on the date when it is accepted by the Licensee as set forth in Article 3.1.

4.2 TERM

The Agreement shall remain in force for the entire legal term of protection of the economic rights over the Software.

Article 5 - SCOPE OF RIGHTS GRANTED

The Licensor hereby grants to the Licensee, who accepts, the following rights over the Software for any or all use, and for the term of the Agreement, on the basis of the terms and conditions set forth hereinafter.

Besides, if the Licensor owns or comes to own one or more patents protecting all or part of the functions of the Software or of its components, the Licensor undertakes not to enforce the rights granted by these patents against successive Licensees using, exploiting or modifying the Software. If these patents are transferred, the Licensor undertakes to have the transferees subscribe to the obligations set forth in this paragraph.

5.1 RIGHT OF USE

The Licensee is authorized to use the Software, without any limitation as to its fields of application, with it being hereinafter specified that this comprises:

1. permanent or temporary reproduction of all or part of the Software by any or all means and in any or all form.

2. loading, displaying, running, or storing the Software on any or all medium.

3. entitlement to observe, study or test its operation so as to determine the ideas and principles behind any or all constituent elements of said Software. This shall apply when the Licensee carries out any or all loading, displaying, running, transmission or storage operation as regards the Software, that it

is entitled to carry out hereunder.

5.2 ENTITLEMENT TO MAKE CONTRIBUTIONS

The right to make Contributions includes the right to translate, adapt, arrange, or make any or all modifications to the Software, and the right to reproduce the resulting software.

The Licensee is authorized to make any or all Contributions to the Software provided that it includes an explicit notice that it is the author of said Contribution and indicates the date of the creation thereof.

5.3 RIGHT OF DISTRIBUTION

In particular, the right of distribution includes the right to publish, transmit and communicate the Software to the general public on any or all medium, and by any or all means, and the right to market, either in consideration of a fee, or free of charge, one or more copies of the Software by any means.

The Licensee is further authorized to distribute copies of the modified or unmodified Software to third parties according to the terms and conditions set forth hereinafter.

5.3.1 DISTRIBUTION OF SOFTWARE WITHOUT MODIFICATION

The Licensee is authorized to distribute true copies of the Software in Source Code or Object Code form, provided that said distribution complies with all the provisions of the Agreement and is accompanied by:

1. a copy of the Agreement,
2. a notice relating to the limitation of both the Licensor's warranty and liability as set forth in Articles 8 and 9,

and that, in the event that only the Object Code of the Software is redistributed, the Licensee allows future Licensees unhindered access to the full Source Code of the Software by indicating how to access it, it being understood that the additional cost of acquiring the Source Code shall not exceed the cost of transferring the data.

5.3.2 DISTRIBUTION OF MODIFIED SOFTWARE

When the Licensee makes a Contribution to the Software, the terms and conditions for the distribution of the resulting Modified Software become subject to all the provisions of this Agreement.

The Licensee is authorized to distribute the Modified Software, in source code or object code form, provided that said distribution complies with all the provisions of the Agreement and is accompanied by:

1. a copy of the Agreement,
2. a notice relating to the limitation of both the Licensor's warranty and liability as set forth in Articles 8 and 9,

and that, in the event that only the object code of the Modified Software is redistributed, the Licensee allows future Licensees unhindered access to the full source code of the Modified Software by indicating how to access it, it being understood that the additional cost of acquiring the source code shall not exceed the cost of transferring the data.

5.3.3 DISTRIBUTION OF EXTERNAL MODULES

When the Licensee has developed an External Module, the terms and conditions of this Agreement do not apply to said External Module, that may be distributed under a separate license agreement.

5.3.4 COMPATIBILITY WITH THE GNU GPL

The Licensee can include a code that is subject to the provisions of one of the versions of the GNU GPL in the Modified or unmodified Software, and distribute that entire code under the terms of the same version of the GNU GPL.

The Licensee can include the Modified or unmodified Software in a code that is subject to the provisions of one of the versions of the GNU GPL, and distribute that entire code under the terms of the same version of the GNU GPL.

Article 6 - INTELLECTUAL PROPERTY

6.1 OVER THE INITIAL SOFTWARE

The Holder owns the economic rights over the Initial Software. Any or all use of the Initial Software is subject to compliance with the terms and conditions under which the Holder has elected to distribute its work and no one shall be entitled to modify the terms and conditions for the distribution of said Initial Software.

The Holder undertakes that the Initial Software will remain ruled at least by this Agreement, for the duration set forth in Article 4.2.

6.2 OVER THE CONTRIBUTIONS

The Licensee who develops a Contribution is the owner of the intellectual property rights over this Contribution as defined by applicable law.

6.3 OVER THE EXTERNAL MODULES

The Licensee who develops an External Module is the owner of the intellectual property rights over this External Module as defined by applicable law and is free to choose the type of agreement that shall govern its distribution.

6.4 JOINT PROVISIONS

The Licensee expressly undertakes:

1. not to remove, or modify, in any manner, the intellectual property notices attached to the Software;
2. to reproduce said notices, in an identical manner, in the copies of the Software modified or not.

The Licensee undertakes not to directly or indirectly infringe the intellectual property rights of the Holder and/or Contributors on the Software and to take, where applicable, vis-à-vis its staff, any and all measures

required to ensure respect of said intellectual property rights of the Holder and/or Contributors.

Article 7 - RELATED SERVICES

7.1 Under no circumstances shall the Agreement oblige the Licensor to provide technical assistance or maintenance services for the Software.

However, the Licensor is entitled to offer this type of services. The terms and conditions of such technical assistance, and/or such maintenance, shall be set forth in a separate instrument. Only the Licensor offering said maintenance and/or technical assistance services shall incur liability therefor.

7.2 Similarly, any Licensor is entitled to offer to its licensees, under its sole responsibility, a warranty, that shall only be binding upon itself, for the redistribution of the Software and/or the Modified Software, under terms and conditions that it is free to decide. Said warranty, and the financial terms and conditions of its application, shall be subject of a separate instrument executed between the Licensor and the Licensee.

Article 8 - LIABILITY

8.1 Subject to the provisions of Article 8.2, the Licensee shall be entitled to claim compensation for any direct loss it may have suffered from the Software as a result of a fault on the part of the relevant Licensor, subject to providing evidence thereof.

8.2 The Licensor's liability is limited to the commitments made under this Agreement and shall not be incurred as a result of in particular: (i) loss due the Licensee's total or partial failure to fulfill its obligations, (ii) direct or consequential loss that is suffered by the Licensee due to the use or performance of the Software, and (iii) more generally, any consequential loss. In particular the Parties expressly agree that any or all pecuniary or business loss (i.e. loss of data, loss of profits, operating loss, loss of customers or orders, opportunity cost, any disturbance to business activities) or any or all legal proceedings instituted against the Licensee by a third party, shall constitute consequential loss and shall not provide entitlement to any or all compensation from the Licensor.

Article 9 - WARRANTY

9.1 The Licensee acknowledges that the scientific and technical state-of-the-art when the Software was distributed did not enable all possible uses to be tested and verified, nor for the presence of possible defects to be detected. In this respect, the Licensee's attention has been drawn to the risks associated with loading, using, modifying and/or developing and reproducing the Software which are reserved for experienced users.

The Licensee shall be responsible for verifying, by any or all means, the suitability of the product for its requirements, its good working order, and for ensuring that it shall not cause damage to either persons or properties.

9.2 The Licensor hereby represents, in good faith, that it is entitled to grant all the rights over the Software (including in particular the rights set forth in Article 5).

9.3 The Licensee acknowledges that the Software is supplied "as is" by the Licensor without any other express or tacit warranty, other than that provided for in Article 9.2 and, in particular, without any warranty as to its commercial value, its secured, safe, innovative or relevant nature.

Specifically, the Licensor does not warrant that the Software is free from any error, that it will operate

without interruption, that it will be compatible with the Licensee's own equipment and software configuration, nor that it will meet the Licensee's requirements.

9.4 The Licensor does not either expressly or tacitly warrant that the Software does not infringe any third party intellectual property right relating to a patent, software or any other property right. Therefore, the Licensor disclaims any and all liability towards the Licensee arising out of any or all proceedings for infringement that may be instituted in respect of the use, modification and redistribution of the Software. Nevertheless, should such proceedings be instituted against the Licensee, the Licensor shall provide it with technical and legal assistance for its defense. Such technical and legal assistance shall be decided on a case-by-case basis between the relevant Licensor and the Licensee pursuant to a memorandum of understanding. The Licensor disclaims any and all liability as regards the Licensee's use of the name of the Software. No warranty is given as regards the existence of prior rights over the name of the Software or as regards the existence of a trademark.

Article 10 - TERMINATION

10.1 In the event of a breach by the Licensee of its obligations hereunder, the Licensor may automatically terminate this Agreement thirty (30) days after notice has been sent to the Licensee and has remained ineffective.

10.2 A Licensee whose Agreement is terminated shall no longer be authorized to use, modify or distribute the Software. However, any licenses that it may have granted prior to termination of the Agreement shall remain valid subject to their having been granted in compliance with the terms and conditions hereof.

Article 11 - MISCELLANEOUS

11.1 EXCUSABLE EVENTS

Neither Party shall be liable for any or all delay, or failure to perform the Agreement, that may be attributable to an event of force majeure, an act of God or an outside cause, such as defective functioning or interruptions of the electricity or telecommunications networks, network paralysis following a virus attack, intervention by government authorities, natural disasters, water damage, earthquakes, fire, explosions, strikes and labor unrest, war, etc.

11.2 Any failure by either Party, on one or more occasions, to invoke one or more of the provisions hereof, shall under no circumstances be interpreted as being a waiver by the interested Party of its right to invoke said provision(s) subsequently.

11.3 The Agreement cancels and replaces any or all previous agreements, whether written or oral, between the Parties and having the same purpose, and constitutes the entirety of the agreement between said Parties concerning said purpose. No supplement or modification to the terms and conditions hereof shall be effective as between the Parties unless it is made in writing and signed by their duly authorized representatives.

11.4 In the event that one or more of the provisions hereof were to conflict with a current or future applicable act or legislative text, said act or legislative text shall prevail, and the Parties shall make the necessary amendments so as to comply with said act or legislative text. All other provisions shall remain effective. Similarly, invalidity of a provision of the Agreement, for any reason whatsoever, shall not cause the Agreement as a whole to be invalid.

11.5 LANGUAGE

The Agreement is drafted in both French and English and both versions are deemed authentic.

Article 12 - NEW VERSIONS OF THE AGREEMENT

12.1 Any person is authorized to duplicate and distribute copies of this Agreement.

12.2 So as to ensure coherence, the wording of this Agreement is protected and may only be modified by the authors of the License, who reserve the right to periodically publish updates or new versions of the Agreement, each with a separate number. These subsequent versions may address new issues encountered by Free Software.

12.3 Any Software distributed under a given version of the Agreement may only be subsequently distributed under the same version of the Agreement or a subsequent version, subject to the provisions of Article 5.3.4.

Article 13 - GOVERNING LAW AND JURISDICTION

13.1 The Agreement is governed by French law. The Parties agree to endeavor to seek an amicable solution to any disagreements or disputes that may arise during the performance of the Agreement.

13.2 Failing an amicable solution within two (2) months as from their occurrence, and unless emergency proceedings are necessary, the disagreements or disputes shall be referred to the Paris Courts having jurisdiction, by the more diligent Party.

1 CeCILL stands for Ce(a) C(nrs) I(nria) L(ogiciel) L(ibre)

Version 2.0 dated 2006-09-05.
