# Supplementary material for: Implementation of a Model of Bodily Fluids Regulation
Source: Acta Biotheor. 2015 May 3;63(3):269–82. doi: 10.1007/s10441-015-9250-3 (PMC4531145; doi:10.1007/s10441-015-9250-3)
Supplement: Supplementary file 4 — Supplementary material 4 (pdf 37 KB) [file 10441_2015_9250_MOESM4_ESM.pdf]

```
;file: "ESM_03.pdf": Simulation of intravenous infusion of physiological saline at a
;      rate of 1000ml per 5 minutes.
;We release this model code under the CeCill free software license agreement.
;
;This file is Supplementary Material for the following article:
;Title: Implementation of a model of bodily fluids regulation
;Journal: Acta Biotheoretica
;Authors: Julie Fontecave-Jallon
;         University Joseph Fourier-Grenoble, CNRS
;         TIMC-IMAG Laboratory
;         CNRS UMR 5525, PRETA team
;         Grenoble, F-38041, France
;         email: <Julie.Fontecave@imag.fr>
;     and
;        S. Randall Thomas
;        IR4M UMR8081 CNRS and University Paris-Sud
;        Orsay, France
;        email: <stephen-randall.thomas@u-psud.fr>
;
;*****************************************************************
; Simulation of intravenous infusion of physiological saline at a rate of 1000ml per 5 minutes
; Figure 10 of Ikeda et al. (1979)
;
; Simulation during 3 hours of the responses of body fluid and kidney parameters to
;   intravenous normal saline infusion, solution of 0.9% w/v of NaCl, containing
;   154 mEq/l of Na+ and Cl-, at a rate of 200ml per minute during 5 minutes.
; QVIN=0 is replaced by QVIN=IF (TIME > 5 AND TIME < 10) THEN 0.2 ELSE 0
; YCLI=0.1328  is replaced by YCLI=IF (TIME > 5 AND TIME < 10) THEN 154*0.2 ELSE 0.1328
; YNIN=0.12 is replaced by YNIN=IF (TIME > 5 AND TIME < 10) THEN 154*0.2 ELSE 0.12
;*****************************************************************

METHOD RK4

STARTTIME = 0
STOPTIME=180 {minutes}
DT = 0.001
DTOUT=0.2

;*****************************************************************
; BLOCK 1 - CARDIOVASCULAR SYSTEM
; Input = VB
; Output = QCO, PAS, PVS, PVP, PAP
;*****************************************************************
; constant parameters
RTOT=20     ; mmHg.min/l
KR=0.3      ;
RTOP=3      ; mmHg.min/l
KL=0.2      ;
DEN=1           ;

QCO0=VB*DEN ; l/min
QCO=QCO0+1      ; l/min

PAS=20+RTOT*QCO0            ; mmHg
PVS=MAX(0,-10.33+QCO0/KR)       ; mmHg
PAP=8+RTOP*QCO0             ; mmHg
PVP=MAX(-16+QCO0/KL,0)      ; mmHg

;*****************************************************************
; BLOCK 2 - RESPIRATION
; Input = STBC, QCO, VTW
; Output = DCLA, PCOA, XCO3, PHA
;*****************************************************************
; constant parameters
XHB=15          ; g/dl
FO2I=0.21           ;
FCOI=0          ;
PBA=760         ; mmHg
PBL=PBA-47          ; mmHg
VAL=3               ; l
MRCO=0.2318     ; l(STPD)/min
MRO2=0.2591     ; l(STPD)/min

; equations (3) to (8)
d/dt(FCOA)=(VI*(FCOI-FCOA)+863/(PBA-47)*QCO*(UCOV-UCOA))/VAL
d/dt(FO2A)=(VI*(FO2I-FO2A)+863/(PBA-47)*QCO*(UO2V-UO2A))/VAL
d/dt(UCOV)=(MRCO+QCO*(UCOA-UCOV))/VTW
d/dt(UO2V)=(-MRO2+QCO*(UO2A-UO2V))/VTW
init(FCOA)=0.0561       ;
init(FO2A)=0.1473       ;
```

```
init(UCOV)=0.6075       ; l(STPD)/l.blood
init(UO2V)=0.1515       ; l(STPD)/l.blood

UCOA=6.732*10^-4*PCOA+0.02226*XCO3  ; l(STPD)/l.blood
UO2A=3.168*10^-5*PO2A+UHBO          ; l(STPD)/l.blood

; equation (1)
d/dt(VI)=(VR*VI0-VI)/2
init(VI)=5      ; l/min

; equation (2) and  F21
k1=IF PHA<=7.4 THEN 0.22 ELSE 0.0258
k6=IF PHA<=7.4 THEN -12.734 ELSE -5.003
k3=0.58
k4=3.496
k2=IF PCOA>40 THEN 1 ELSE 0.0396
k5=IF PCOA>40 THEN -32.08 ELSE 160.11

H=10^(9-PHA)
VR=k1*H+k2*(k3+k4/(PO2A-32))*(PCOA+k5)+k6
LIMIT VR >= 0
VI0=5           ; l

; F23
f=(1-exp(-PO2A*g))^2
g=0.0066815*PHA^3-0.10098*PHA^2+0.44921*PHA-0.454
UHBO=UHB*f          ; l.O2(STPD)/l.blood
DCLA=XCO3-STBC      ; mEq/l
UHB=XHB/75          ; l.O2(STPD)/l.blood
PCOA=FCOA*(PBA-47)  ; mmHg
PO2A=FO2A*(PBA-47)  ; mmHg

; equation (9) and F22
PHA=6.1+log10(XCO3/(0.03*PCOA))     ;

; F24
d/dt(XCO3)= (STBC-(0.527*XHB+3.7)*(PHA-7.4)+0.375*(UHB-UHBO)/0.02226-XCO3)
init(XCO3)=24       ; mEq/l

;*****************************************************************
; BLOCK 3 - EXTRACELLULAR SPACE
; Input = PAS, PVS, QIC, QWU
; Output = PPCO, VB; VEC, XPP
;*****************************************************************
; constant parameters
QIN=0.001       ; l/min
;QVIN=0     ; l/min
QVIN=IF time>5 AND time<=10 THEN 0.2 ELSE 0
QIWL=0.0005 ; l/min
QMWP=0.0005 ; l/min
QLF0=0.002      ; l/min
VIF0=8.8        ; l
CFC=0.007       ; l/min/mmHg
VRBC=1.8        ; l
CRAV=5.93       ;
VB=VRBC+VP      ; l
HT=VRBC/VB      ;
VEC=VP+VIF      ; l

d/dt(VIN)=QIN-(VIN/10)
init (VIN)=0.01     ; l

d/dt(VIF)=QCFR-QLF-QIC
init (VIF)=VIF0     ; l

PC=(CRAV*PVS+PAS)/(1+CRAV)      ; mmHg

d/dt(ZPP)=YPLF-YPLG-YPLV-YPLC
init(ZPP)=154       ; g

XPP=ZPP/VP          ; g/l
YPLC=QPLC*(XPP-XPIF)    ; g/min
XPIF=ZPIF/VIF       ; g/l

d/dt(ZPIF)=YPLC-YPG-YPLF
init(ZPIF)=176      ; g

YPLF=XPIF*QLF           ; g/min
YPLV=XPP*0.00047-0.0329 ; g/min
YPLG=0.00023*(XPP-ZPLG)     ; g/min
```

```
d/dt(ZPLG)=(XPP-ZPLG)/24
init(ZPLG)=70           ; g

YPG=0.0057*(XPIF-ZPG)   ;g/min

d/dt(ZPG)=(XPIF-ZPG)/150
init (ZPG)=20           ; g

; equation (10)
VP_plus=VIN/10+QVIN+QMWP+QLF
; equation (11)
VP_minus=QIWL+QWU+QCFR
; equation (12)
d/dt(VP)=VP_plus-VP_minus
init(VP)=2.2            ; l
; equation (13)
QCFR=CFC*Pf         ; l/min
; equation (14)
Pf=PC-PPCO-PIF+PICO     ; mmHg

; F31      ; NB: no line breaks in the equation for the actual program
x=VIF/VIF0          ;
PIF=IF (x<=0.9) THEN (-15) ELSE IF (x>0.9 AND x<=1) THEN (87*x-93.3) ELSE
        IF (x>1 AND x<=2) THEN (-6.3*(2-x)^10) ELSE (x-2)   ; mmHg
; F32
QLF=QLF0*(24/(1+exp(-0.4977*PIF)))  ; l/min
; F33
QPLC=2.768*10^-6*PC^2               ; l/min
; F34
PPCO=0.4*XPP                    ; mmHg
; F35
PICO=0.25*XPIF                  ; mmHg

;******************************************************************
; BLOCK 4 - INTRACELLULAR SPACE and ELECTROLYTES
; Input = GFR, PHA, VEC, YKU, YNU
; Output = QSMP, QIC, VTW, XKE, XNE, YGLU, YHI, YMNU, YNU
;******************************************************************
; constant parameters
CSM=0.0003      ; l≤/mEq/min
;YNIN=0.12      ; mEq/min
YNIN=IF time>5 AND time<=10 THEN 30.8 ELSE 0.12
CKEI=0.001      ;
YKIN=0.047      ; mEq/min
XGLO=108        ; mg/dl
YINS=0          ; unit/min
YGLI=0          ; mg/min
YMNI=0          ; mM/min
YURI=0.15           ; mM/min
CGL1=1          ;
CGL2=1          ;
CGL3=0.03           ;
CHEI=5          ;
CBFI=10^-9

; equation (15)
d/dt(ZNE)=YNIN-YNU+YHI
init(ZNE)=1540      ; mEq

; equation (16)
d/dt(ZKE)=YKIN-YKU-y4
init(ZKE)=49.5      ; mEq

d/dt(ZKI)=y4
init(ZKI)=2800      ; mEq
y4=z4+CKEI*(2800*F41-ZKI)
; F41
F41=1+0.5*log10(XKE/(56.744-7.06*PHA))
; F42
YGLU=IF XGLE*GFR<0.65 THEN 0 ELSE  XGLE*GFR-0.65    ; mg/min
XNE=ZNE/VEC     ; mEq/l
XKI=ZKI/VIC     ; mEq/l
XKE=ZKE/VEC     ; mEq/l

d/dt(ZHI)=YHI
YHI=CHEI*(0.4-PHA+PHI)
init(ZHI)=100

PHI=-log10(CBFI*ZHI)
```

```
d/dt(YINT)=1/1.50*(XGLE-XGLO/18-YINT)
init(YINT)=0

YGLS=CGL1*YINT+CGL2*YINS
z4=CGL3*YGLS

d/dt(ZGLE)=YGLI/180-YGLS-YGLU
init(ZGLE)=66       ; mg

XGLE=ZGLE/VEC       ; mg/l
OSMP=(XNE+XKE)*1.86+XGLE+XURE+XMNE+9.73     ; mOsm/l

d/dt(ZMNE)=YMNI-YMNU
init(ZMNE)=0        ; mM

d/dt(ZURE)=YURI-YURU
init(ZURE)=77.5     ; mM

XMNE=ZMNE/VEC       ; mM/l
XURE=ZURE/VTW       ; mM/l
VTW=VEC+VIC         ; l
YMNU=1*GFR*XMNE     ; mM/min
YURU=XURE*GFR*0.6   ; mM/min

d/dt(VIC)=QIC
init(VIC)=20        ; l
QIC=CSM*((-XNE-XKE)-XGLE+(10.5+XKI))    ; l/min

;*****************************************************************
; BLOCK 5 - KIDNEY 2
; Input = DCLA,GFR,PCOA,VEC,XCO3,XKE,XNE,XPP,YHI,YKU,YNH4,YNU, STPG
; Output = STBC, YCO3, YORG, YPO4
;*****************************************************************
; constant parameters
YCAI=0.007      ; mEq/min
;YCLI=0.1328    ; mEq/min
YCLI=IF time>5 AND time<=10 THEN 30.8 ELSE 0.1328
YMGI=0.008      ; mEq/min
YOGI=0.01       ; mM/min
YPOI=0.025  ; mM/min
YSOI=0.02       ; mEq/min

; F50
F50=-PCOA/120+4/3
; F51  ; NB: no line breaks in the equation for the actual program
YCO3=IF XCO3*GFR*F50<=2 THEN 0 ELSE IF (XCO3*GFR*F50>2 AND XCO3*GFR*F50<=4)
     THEN 0.1638*( XCO3*GFR*F50-2)^2.61 ELSE  XCO3*GFR*F50-3    ; mEq/min
; F52
YCA=IF XCAE*GFR<0.493 THEN 0 ELSE  XCAE*GFR-0.493       ; mEq/min
; F53
YMG=IF XMGE*GFR<0.292 THEN 0 ELSE  XMGE*GFR-0.292       ; mEq/min
; F54
YSO4=IF XSO4*GFR<0.08 THEN 0 ELSE  XSO4*GFR-0.08        ; mEq/min
; F55
YPO4=IF XPO4*GFR<=0.11 THEN 5/22*XPO4*GFR ELSE  XPO4*GFR-0.085  ;mM/min
; F56
YORG=IF XOGE*GFR<=0.6 THEN XOGE*GFR/60 ELSE  XOGE*GFR/3-0.19    ; mM/min

d/dt(ZCAE)=YCAI-YCA
init(ZCAE)=55       ; mEq

d/dt(ZMGE)=YMGI-YMG
init(ZMGE)=33       ; mEq

d/dt(ZSO4)=YSOI-YSO4
init(ZSO4)=11       ; mEq

d/dt(ZPO4)=YPOI-YPO4
init(ZPO4)=12.1     ; mM

d/dt(ZOGE)=YOGI-YORG
init(ZOGE)=66       ; mM

d/dt(ZCLE)=YCLI-YCLU
init(ZCLE)=1144     ; mEq

XCAE=ZCAE/VEC       ; mEq/l
XMGE=ZMGE/VEC       ; mEq/l
XSO4=ZSO4/VEC       ; mEq/l
```

```
XPO4=ZPO4/VEC        ; mM/l
XOGE=ZOGE/VEC        ; mM/l
XCLE=ZCLE/VEC        ; mEq/l
XCLA=XCLE-DCLA       ; mEq/l
STBC=XCAE+XMGE-XSO4-1.8*XPO4-XOGE-XCLE+XNE+XKE-0.2214*XPP    ; mEq/l
YCLU=MAX(0,YNU+YKU-STPG+YNH4-YCO3+YCA+YMG-YSO4)              ; mEq/min

;*****************************************************************
; BLOCK 6 - KIDNEY 1
; Input = ADH, ALD, GFR, OSMP, PHA, THDF, XKE, XNE, YCO3, YGLU, YORG,
;             YPO4, YMNU, YURU
; Output = STPG, QWU, YKU, YNH, YNH4,YNU
;*****************************************************************
; constant parameters
CPRX=0.2        ;
YNH0=0.024      ; mEq/min
YTA0=0.0068 ; mEq/min

; F61
YNH4=YNH0*(-0.5*PHU1+4) ; mEq/min
; F62
F62=YTA0*(-2.5*PHA1+19.5)
; F63
YTA1=IF PHU2<=4 THEN 0 ELSE IF (PHU2>4 AND PHU2<=5) THEN F62*(PHU2-4) ELSE F62  ; mEq/min

d/dt(PHU2)=(PHU-PHU2)
init(PHU2)=6

; F64
STPO=YPO4*(1+1/(1+10^(6.8-PHA)))        ; mM/min

; F65 ; NB: no line breaks in the equation for the actual program
PHU=-log10((-((10^-4.3+10^-6.8)*(STPG-YPO4-(1/(1+10^(PHA-4.3)))*YORG)
    -10^-6.8*YPO4-10^-4.3*YORG) + (((10^-4.3+10^-6.8)*(STPG-YPO4-
    (1/(1+10^(PHA-4.3)))*YORG)-10^-6.8*YPO4-10^-4.3*YORG)^2-4
    *(STPG-YPO4-(1/(1+10^(PHA-4.3)))*YORG)*((10^-4.3*10^-6.8)
    *((STPG-YPO4-(1/(1+10^(PHA-4.3)))*YORG)
    -YPO4-YORG)))^(0.5))/2/(STPG-YPO4-(1/(1+10^(PHA-4.3)))*YORG))

STPG=MAX(0,STPO+YORG-YTA)
YTA=YTA1+0.009+ALD*0.001

d/dt(PHA1)=(PHA-PHA1)/200
init(PHA1)=7.4

d/dt(PHU1)=(PHU-PHU1)/300
init(PHU1)=6

YNOD=MAX(0,YTA1+YNH4-YCO3)                  ; mEq/min
YNU=MAX(YND*0.116-YNOD,0)                   ; mEq/min
GFR1_6=THDF*GFR*CPRX                        ; l/min
YND=XNE*GFR1_6*0.5*0.9-ALD*0.09             ; mEq/min
YKU=0.39*YKD                            ; mEq/min
YKD=ALD*0.018*XKE+0.9*0.5*GFR1_6*XKE            ; mEq/min
OSMU=(YGLU+YURU+YMNU+1.86*(YKU+YNU))/QWU        ; mOsm/l
QWD=(YGLU+YURU+YMNU+(YND+YKD)*1.86+0.32)/OSMP   ; l/min
QWU=QWD-QWD*0.9*ADH                     ; l/min
YNH=0.5*GFR1_6*XNE                      ; mEq/min

;*****************************************************************
; BLOCK 7 - CONTROLLER of RENAL FUNCTION
; Input = OSMP, PAS, PPCO, PVP, VEC, XKE, XNE,YNH
; Output = ADH, ALD, GFR, THDF
;*****************************************************************
; constant parameters
CKAL=0.5        ;
CNAL=0.1        ;
COAD=0.5        ;
CPAD=1      ;
CPAL=0.01       ;
CPVL=0.1        ;
GFR0=0.1        ; l/min
ACTH=1      ;
VEC0=11     ; l
TADH=30     ; min
TALD=30     ; min

; F71
ADH=1.1/(1+exp(-0.5*(ADH0+4.605)))
; F72
```

```
ALD=10/(1+exp(-0.4394*(ALD0-5)))
; F73
THDF=IF PPCO<=28 THEN -5*(PPCO/28-1)+1 ELSE 1       ; l
; F74  ; NB: no line breaks in the equation for the actual program
GFR1=IF PAS<40 THEN 0 ELSE IF (PAS>=40 AND PAS <80) THEN 0.02*PAS-0.8
        ELSE IF (PAS>=80 AND PAS<100) THEN -0.0005*(PAS-100)^2+1 ELSE 1
GFR=GFR1*GFR0*VEC/VEC0      ; l/min

d/dt(ALD0)=(ALD1-ALD0)/TALD
init(ALD0)=0

d/dt(ADH0)=(z7-ADH0)/TADH
init(ADH0)=0
z7=(OSMP-287)*COAD-(PVP-4)*CPAD

ALD1=(ACTH-1)*1+(XKE-4.5)*CKAL-(PVP-4)*CPVL-(YNH-1.4)*CNAL-(PAS-100)*CPAL

DISPLAY QIN,ADH,ALD,STBC,PAS,VIF,OSMP,VIC,VEC,VP,QWU
```
